# Unusual Dual Complications: Bowel Obstruction and Hydronephrosis Secondary to a Large Ovarian Serous Cystadenoma in a Postmenopausal Patient

**DOI:** 10.7759/cureus.87081

**Published:** 2025-06-30

**Authors:** Amber R Jacobson, Hamdan Mallick, Lynnsey M Rebner, Megan Poremba, Anirudha Goparaju

**Affiliations:** 1 General Surgery, Bayhealth Medical Center, Dover, USA; 2 Medicine, Bayhealth Medical Center, Dover, USA; 3 General Surgery, Philadelphia College of Osteopathic Medicine, Philadelphia, USA; 4 Bariatric Surgery, Minimally Invasive Surgery, Bayhealth Medical Center, Sussex, USA

**Keywords:** benign ovarian cysts, giant ovarian tumors, obstructive hydronephrosis, ovarian cyst, serous cystadenoma, small bowel obstruction

## Abstract

Ovarian cystadenomas are common benign tumors that are typically detected early; however, some patients develop large, symptomatic masses. We report the case of a 72-year-old postmenopausal woman who presented with a two-week history of abdominal pain, nausea, vomiting, and right lower quadrant abdominal fullness. The patient had previously undergone hysterectomy and bilateral oophorectomy. CT imaging revealed a large, multi-loculated pelvic mass (19.3 × 17.4 cm) causing small bowel obstruction and right hydroureteronephrosis. An exploratory laparotomy was performed, and histopathological examination confirmed an ovarian serous cystadenoma. This case highlights that even benign ovarian lesions can lead to significant complications, underscoring the need for a multidisciplinary approach with the support of comprehensive imaging and meticulous surgical planning.

## Introduction

Ovarian cystadenomas are some of the most common benign adnexal tumors [1]. Serous cystadenomas account for 16% of all ovarian epithelial neoplasms, represent two-thirds of benign ovarian epithelial tumors, and occur across a broad age spectrum, with a mean age ranging between 40 and 60 years. They are bilateral in 10-20% of cases [2].

Large ovarian serous cystadenomas are rare [3]. In general, annual gynecologic exams and modern imaging techniques, such as transvaginal or transabdominal ultrasound, can help detect benign ovarian neoplasms before the development of a large mass; however, there remains a subset of patients who will still elude early detection [4]. Case reports of patients with large ovarian masses describe a chronic course of abdominal distension and discomfort increasing over one to two years, only presenting and undergoing surgery once symptoms become unbearable [3-9].

In the present case, we describe a 72-year-old postmenopausal female with a history of total abdominal hysterectomy with possible bilateral versus left oophorectomy who presented with small bowel obstruction (SBO) and hydronephrosis. CT Imaging revealed a large, multi-loculated pelvic mass, initially presumed to be a cystic mass of retroperitoneal origin; however, pathology after surgical removal confirmed an ovarian serous cystadenoma.

## Case presentation

A 72-year-old G3P2 female presented to the emergency department of a critical access hospital with a two-week history of abdominal pain, nausea, vomiting, and dysuria that worsened over the last 24 hours. She also reported recent fullness in the right lower quadrant of her abdomen. She had a past medical history of hypertension, chronic obstructive pulmonary disease, type 2 diabetes, and an open total abdominal hysterectomy with what she initially reported as a bilateral oophorectomy for endometriosis performed over 40 years ago.

**Figure 1:**
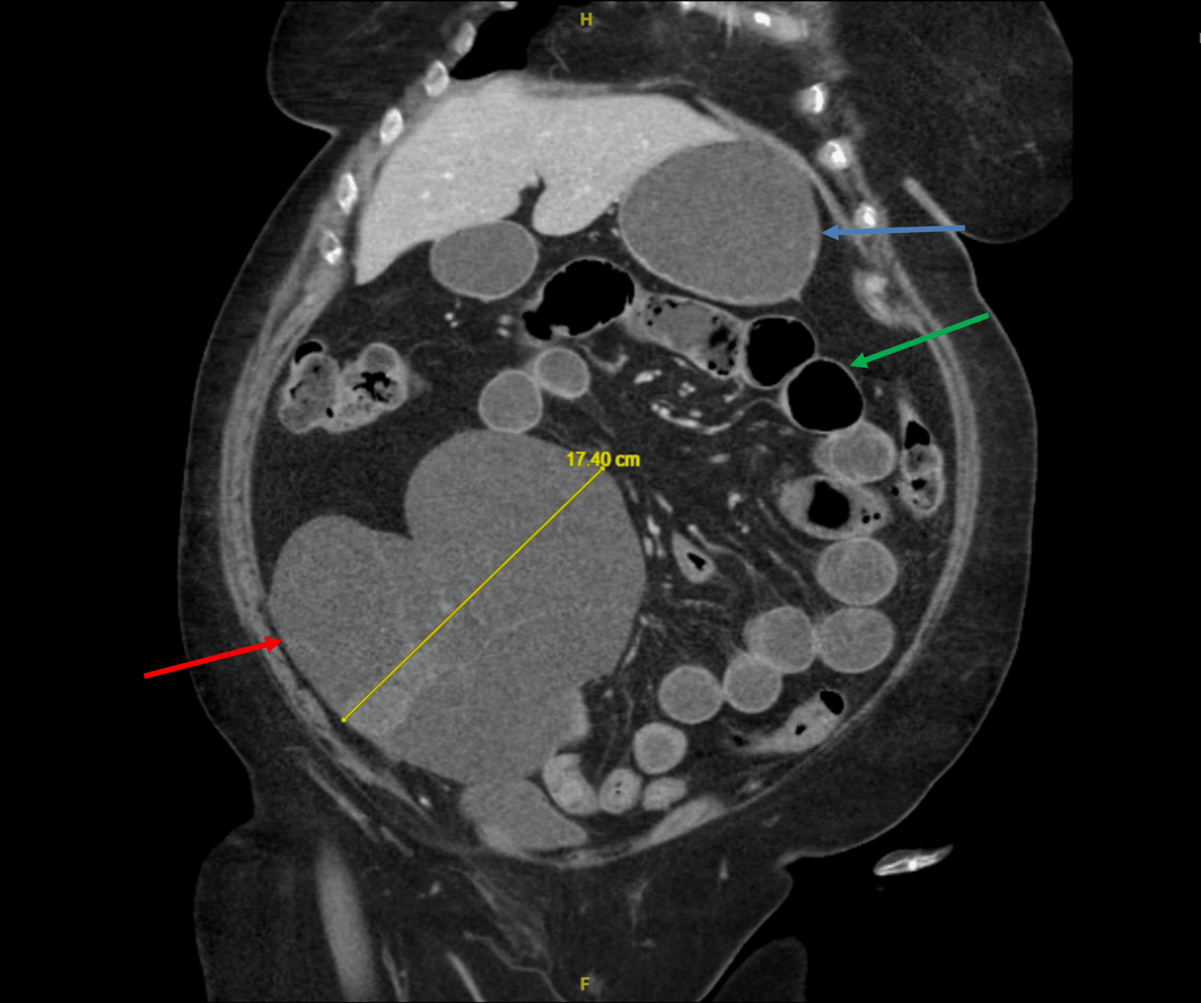
Contrast-enhanced CT scan in the coronal view reveals a large cystic mass (red arrow) present in the right lower quadrant of the abdomen measuring 17.4 cm wide. Fluid-filled stomach (blue arrow) and dilated bowel loops (green arrow).

**Figure 2:**
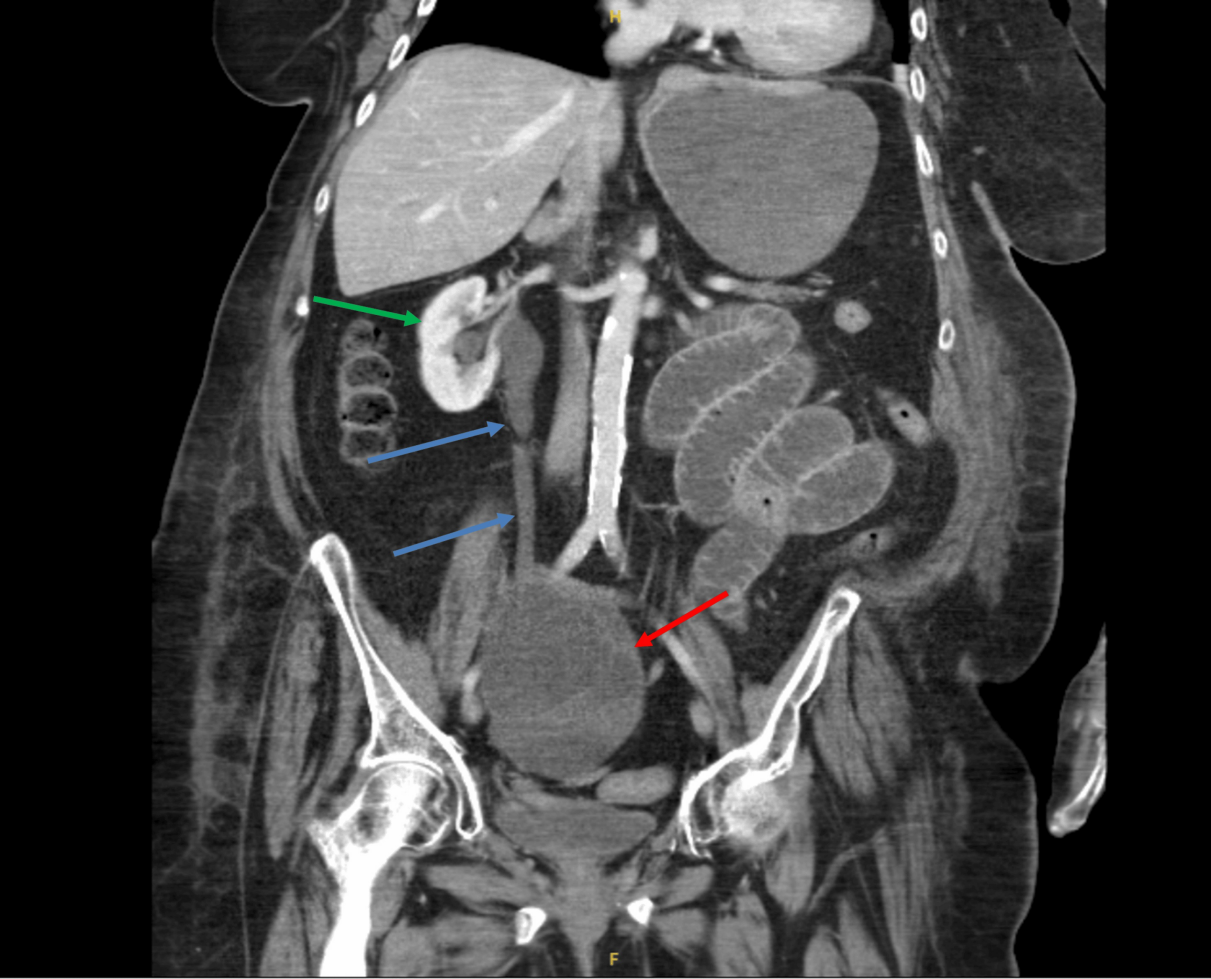
Contrast-enhanced CT scan in the coronal view reveals a cystic mass (red arrow) compressing the right ureter causing right-sided hydroureteronephrosis (bloe arrows). The right kidney shown with the green arrow.

**Figure 3:**
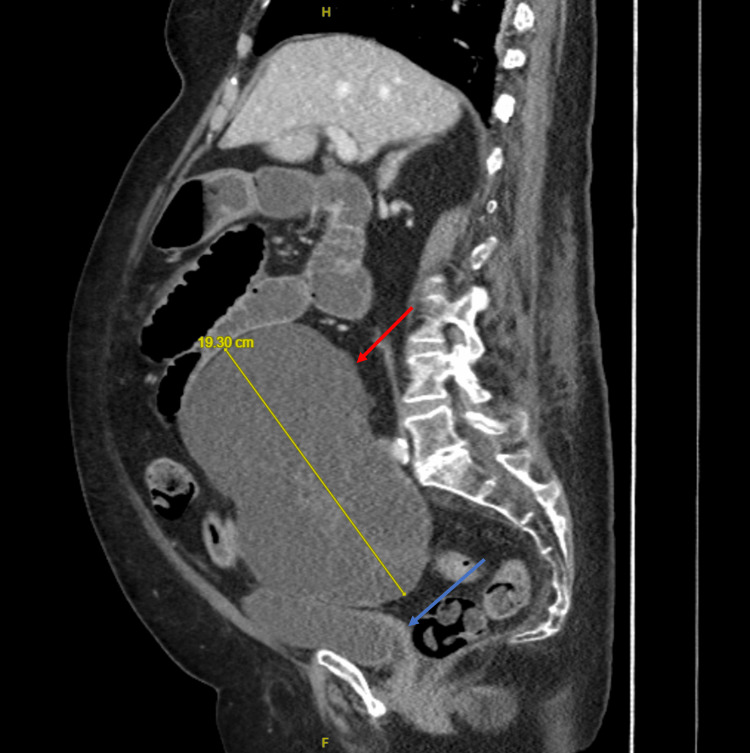
Contrast-enhanced CT scan in the sagittal view reveals a large cystic mass (red arrow) measuring 19.3 cm craniocaudally. The compressed urinary bladder (blue arrow).

On physical examination, she was hemodynamically stable (blood pressure: 137/71 mmHg; pulse: 68 beats/minute; temperature: 98.1°F; saturating 93% on room air) with moderate abdominal distension and right lower quadrant fullness. Laboratory findings were unremarkable (Table 1) with no significant leukocytosis or electrolyte derangement. Preoperative cancer antigen-125 (CA-125) testing was not performed due to low suspicion of ovarian etiology. Intravenous contrast-enhanced CT scan showed a large multi-loculated right lower quadrant abdominal mass measuring 19.3 cm craniocaudally × 17.4 cm transversely. The stomach appeared fluid-filled with dilated loops of bowel and with distal bowel loops decompressed, and no exact transition point was seen (Figure 1). There was also right hydroureteronephrosis without a well-defined transition point, though the ureter appeared tapered and extrinsically compressed in the region of the mass (Figure 2). A mass effect was also seen on the intraperitoneal portion of the urinary bladder (Figure 3). She was initially treated with bowel decompression for five days using a nasogastric tube, which put out 1-3 L of bilious content daily.

**Table 1:** Initial laboratory values in the emergency department.

	Latest reference range and units	Current value
Sodium	136–145 mmol/L	141
Potassium	3.5–5.1 mmol/L	3.8
Chloride	98–107 mmol/L	98
CO^2^	21–32 mmol/L	38 (H)
Anion gap	5.0–15.0 mmol/L	5.0
Blood urea nitrogen	7–18 mg/dL	14
Creatinine serum	0.6–1.0 mg/dL	0.7
Blood urea nitrogen/Creatinine ratio	-	20.0
Estimated glomerular filtration rate	>60 mL/minute/1.73m^2^	>60
Glucose	70–140 mg/dL	77
White blood cell count	4.5–11.0 K/µL	10.2
Red blood cell count	4.00–5.20 M/µL	5.96 (H)
Hemoglobin	12.0–16.0 g/dL	17.0 (H)
Hematocrit	37.0–47.0%	54.0 (H)
Platelets	150–400 K/uL	282

Given her continued unresolved bowel obstruction, she was then taken to the operating room for an exploratory laparotomy. Urology was consulted preoperatively, and a decision was made for same-day proactive placement of bilateral stent insertion to help with identifying landmarks. Cystoscopy was performed at the same time as stent insertion, which did not show abnormalities. Stents were placed without difficulty.

**Figure 4:**
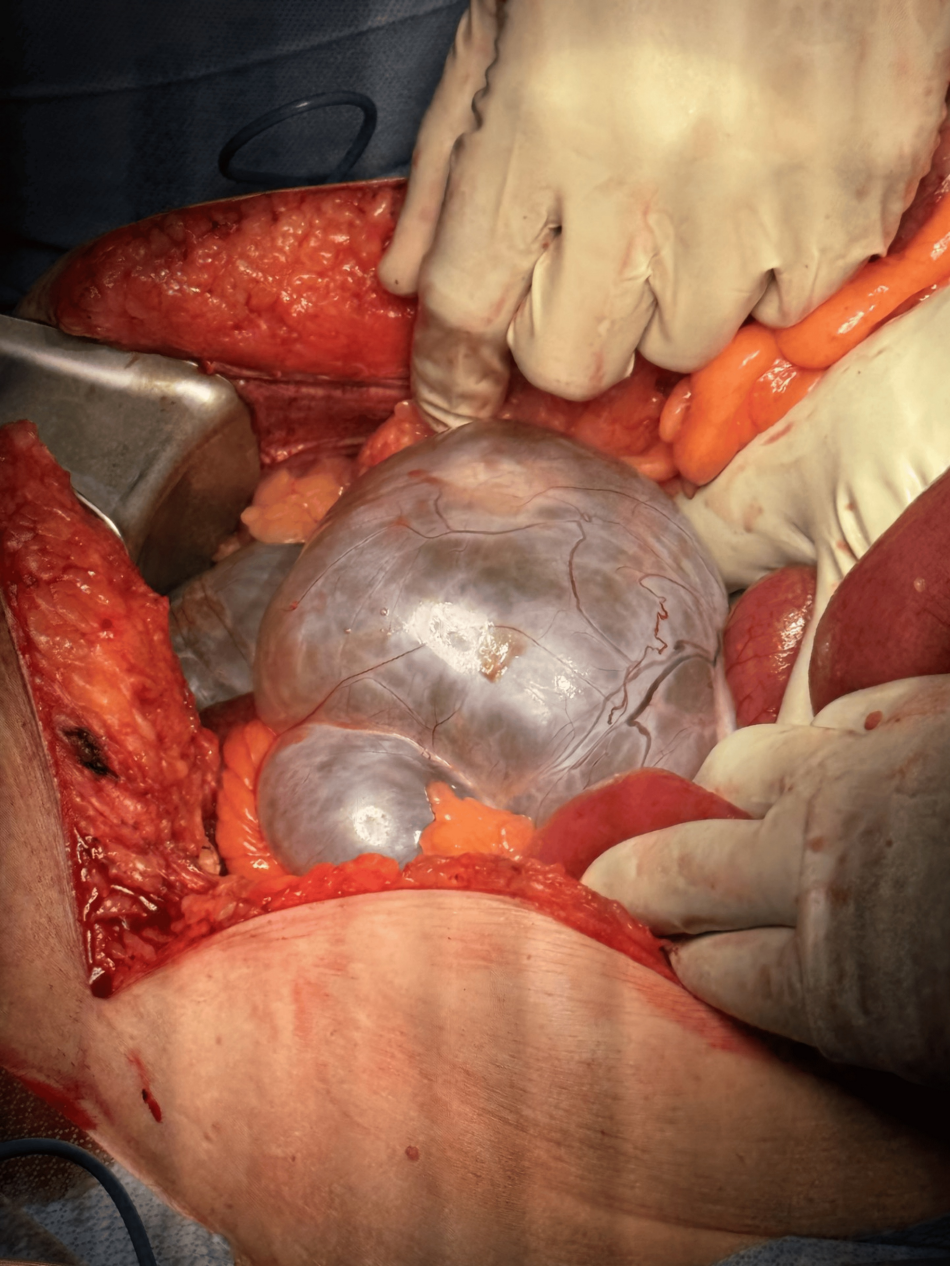
Multi-loculated cystic mass in the right lower quadrant of the abdomen with surrounding distended small bowel.

A vertical midline incision was made from the epigastric to the pubic region. Upon entering the abdomen, there was a blue-tinted, fluid-filled, multi-loculated mass in the right lower quadrant, which was adhered to the small bowel, colon, and retroperitoneum (Figure 4). There was no evidence of ascites or peritoneal metastasis, and the liver and stomach were unremarkable. Given the patient’s stated history of bilateral oophorectomy and retroperitoneal location of the mass, the intraoperative impression was that of a cystic mass of retroperitoneal origin rather than an adnexal lesion. Consequently, the cysts were carefully lanced and evacuated without spillage to facilitate safe dissection off of the surrounding adherent structures.

There was a small solid component posterior to the mass, which was circumferentially removed, and the blood supply appeared to be numerous feeding vessels coming from the common iliac artery and vein, which were detached via Ligasure. There did not appear to be a right fallopian tube present, which was likely removed during her total abdominal hysterectomy. The rest of the cyst was adherent to the surrounding bowel, and this was removed via sharp dissection. The right ureter stent was palpated, and care was taken to avoid injury to the ureter. Once the bowel was freed, we ran the bowel and there was no further evidence of adherent cystic tissue. After hemostasis was achieved, the patient’s abdomen was lavaged, a 15-Fr Jackson-Pratt (JP) drain was left in the right lower quadrant, and the abdominal wall was closed without difficulty. The bilateral ureteral stents and Foley catheter were removed on postoperative day one per urology recommendations, with instructions to follow up with them on an outpatient basis.

**Figure 5:**
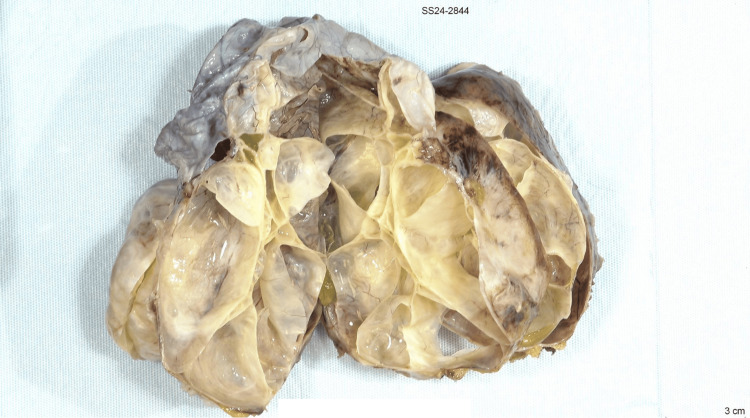
Multi-loculated cystic spaces filled with yellow gelatinous material and straw-colored serous fluid.

**Figure 6:**
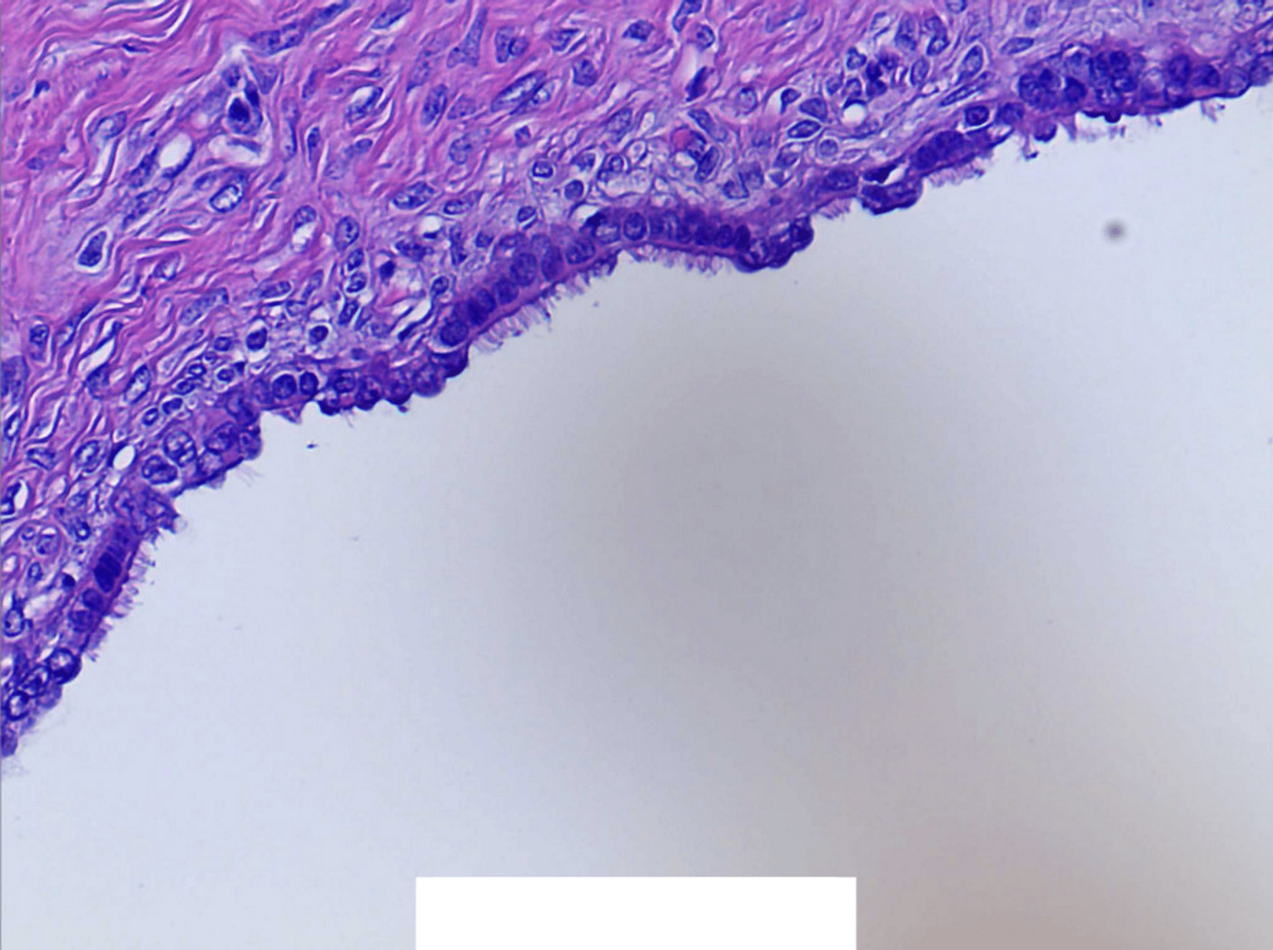
Lining of cysts consistent with epithelial cells with small, bland, round-to-oval nuclei and variable cytoplasm with cilia. The cyst lining cells are positive for CK5/6, Ber-EP4, CK AE1/AE3, ER, PR, Moc-31, PAX8, and WT1. The cyst lining cells are negative for calretinin (40× magnification).

**Figure 7:**
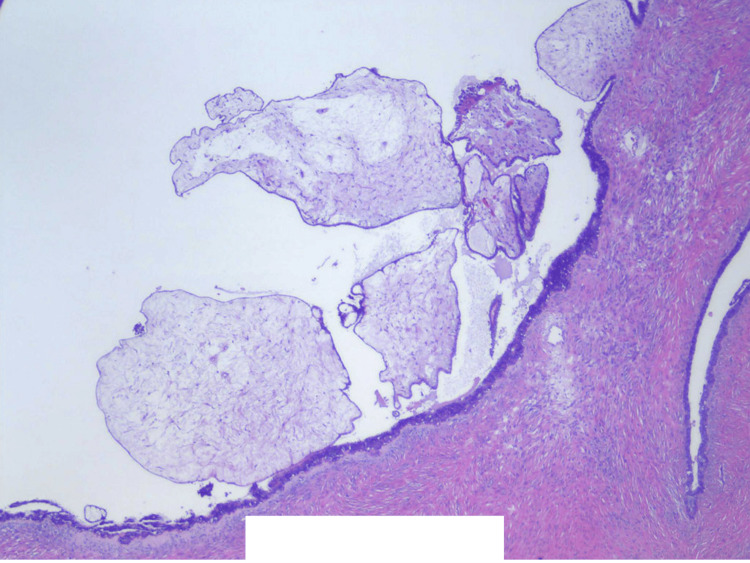
Focal excrescences around cyst lining. The cyst lining cells are positive for CK5/6, Ber-EP4, CK AE1/AE3, ER, PR, Moc-31, PAX8, and WT1. The cyst lining cells are negative for calretinin (20× magnification).

Pathology for the mass showed an ovarian serous cystadenoma. The posterior wall was consistent with fragments of benign fibroconnective tissue. Cytology fluid was negative for malignant cells. Immunohistochemistry staining revealed that the cyst lining cells were positive for CK5/6, Ber-EP4, CK AE1/AE3, ER, PR, Moc-31, PAX8, and WT1, revealing epithelial differentiation. They were negative for calretinin, ruling out mesothelial lineage. See Figures 5-7 for gross and micro pathology.

Postoperatively, the patient experienced a transient ileus that resolved with bowel rest. She continued to void without issue with a urine output of at least 0.5 mL/kg/hour. Her creatinine remained less than 1 mg/dL, and her estimated glomerular filtration rate was greater than 60 mL/minute/1.73m^2^. Her JP drain had minimal serosanguinous output and was discontinued. She resumed a regular diet and was ready to be discharged home on postoperative day six, but waited in the hospital for an additional six days for acute rehabilitation placement. Her total hospital stay was 18 days.

At her one-week follow-up, she reported normal bowel and urinary function. Unfortunately, she was lost to follow-up despite outpatient referrals to urology and medical oncology. No follow-up imaging was performed to assess for hydronephrosis resolution due to this. On postoperative review of pathology, the patient reported that she may have only undergone a left unilateral oophorectomy instead of a bilateral oophorectomy; no operative reports were available to investigate this.

## Discussion

In evaluating intra-abdominal cystic masses, it is essential to consider a broad differential diagnosis, including pelvic endometriosis, intra-abdominal cysts arising from various origins (omentum, mesentery, kidney, liver, pancreas, cystic lymphangiomas, choledochal cyst), ovarian cysts, and various malignancies [6,10]. The spectrum of benign ovarian neoplasms in postmenopausal women is diverse, with mucinous cystadenomas often cited as the most common type in this age group; however, serous cystadenomas are the next most common [1]. Our patient presented with an unusual abdominal mass, initially presumed to be a retroperitoneal cystic mass on exploration, which turned out to be an ovarian serous cystadenoma on postoperative pathology reports.

Serous tumors originate from the invagination of the ovarian surface epithelium, resulting in the secretion of serous fluid [3]. These tumors are usually multilocular and, in some cases, may contain papillary projections. They can also be extraovarian in nature and can even present as an accessory ovary [7,11]. They are commonly small and asymptomatic; documented giant ovarian serous cystadenomas remain a rare entity [3]. Even so, previous case reports have described ovarian cysts of exceptional sizes, including two reports of a 23 kg and 86.5 kg serous cystadenoma mass, and the largest documented being 149 kg [8,9]. Given our patient initially reported a bilateral oophorectomy coupled with the atypical retroperitoneal origin and blood supply of this ovarian cyst seen on laparotomy, there is potential that this may have been an extraovarian cyst.

Although the ovarian mass in this case was smaller than those described in previous reports, its mass effect was sufficient to cause concurrent SBO and hydronephrosis. Complications such as bowel obstruction, hydronephrosis, ovarian torsion, and abdominal compartment syndrome have been documented; however, concurrent SBO and hydronephrosis are seldom reported [6]. There was one case report of a 52-year-old patient with bilateral hydronephrosis due to a mass effect on the kidneys from an ovarian mass, but did not have bowel obstruction symptoms [12]. This may be uncommon because benign serous cystadenomas generally grow slowly, which allows for compensatory mechanisms by surrounding organs. Nevertheless, in a previous case report, a 47-year-old female presented with compartment syndrome after 24 months of increased abdominal distension from an ovarian mass [5]. Another case report described SBO and peritonitis as complications that developed after ovarian cyst removal, with flimsy adhesions from the cyst identified as the cause of the obstruction [13]. In a rarer case report, an ovarian cyst was also seen to herniate through mesenteric defects, causing small bowel volvulus and obstruction [14]. In analyzing these reports, mass effect alone was not the sole cause of bowel obstruction and hydronephrosis, which further underscores the rarity of our patient’s case.

A multi-disciplinary approach was also helpful in this patient’s case. Although there were no gynecologic oncology specialists available at our institution, urology was available for consultation. Given that the patient had adequate urine output and normal kidney function on labs, urology initially opted for conservative management for her right-sided hydronephrosis, but after preoperative planning, agreed to place intraoperative stents to assist in identifying landmarks during exploratory surgery. She continued to have normal voiding and kidney function postoperatively with resolution of her dysuria. Unfortunately, the patient did not have postoperative imaging of her hydronephrosis as she was lost to follow-up.

Management of giant ovarian cystadenomas requires careful preoperative planning. Preoperative aspiration of cystic lesions is generally avoided due to risks of bleeding, infection, and potential spillage of malignant cells; however, there are case reports of cystic decompression via mini laparotomy before resection via laparoscopy [6]. Moreover, intraoperative complications, including hemodynamic instability due to sudden decompression of large intra-abdominal masses, must be anticipated, as evidenced by reports of splanchnic dilatation, venous pooling, and even pulmonary edema following mass removal [4]. Laparotomy is preferred for giant ovarian cystadenomas or masses suspicious for malignancy, although laparoscopy is becoming more favorable [1]. In our case, preoperative nasogastric decompression paired with careful cyst evacuation via laparotomy allowed for successful adhesiolysis of the mass off the small bowel, colon, and retroperitoneum while also maintaining hemodynamic stability.

The use of tumor markers such as CA-125, although not performed during the patient's hospitalization, remains valuable in differentiating malignant from benign epithelial ovarian tumors. Initial CA-125 testing for our patient may have potentially led to an ovarian mass diagnosis before surgery, despite a history of bilateral oophorectomy.

Imaging plays a critical role in evaluating ovarian masses, with pelvic ultrasound being the first diagnostic instrument recommended for assessing adnexal pathology (both transvaginal for better imaging and transabdominal for better tolerance) [1]. Buy et al. showed that ultrasound detected benign serous cystadenomas with a sensitivity of 70% and a specificity of 95%. In the same study, CT identified serous cystadenomas ranging from 3 cm to 20 cm in size, with a sensitivity of 69% and a specificity of 96% [15]. Despite these strengths, CT is sometimes of limited value in diagnosing adnexal masses [2]. Although our patient recovered well, ultrasound may have aided in a preoperative diagnosis.

## Conclusions

This case is notable not only for its occurrence in a postmenopausal patient with a prior history of questionable bilateral versus unilateral oophorectomy and hysterectomy but also for its atypical clinical presentation marked by concurrent bowel obstruction and hydronephrosis. While giant ovarian serous cystadenomas are rare, the unique constellation of complications underscores the importance of comprehensive imaging, surgical planning, and consideration of a broad differential diagnosis. The use of tumor markers and other imaging modalities can also further delineate a cystic mass diagnosis. Lastly, this case displays that even benign ovarian lesions can lead to multi-organ compromise, warranting a tailored and multidisciplinary approach to management.
